# Cancer Screening Behaviors and Associations with Childhood Trauma, Resiliency, and Patient–Provider Relationships: Findings from an Exploratory Study of Appalachian Cervical Cancer Survivors

**DOI:** 10.13023/jah.0501.03

**Published:** 2023-04-01

**Authors:** Dannell Boatman, Stephenie Kennedy-Rea, Lesley Cottrell, Hannah Hazard-Jenkins

**Affiliations:** West Virginia University Cancer Institute, dboatman@hsc.wvu.edu; West Virginia University Cancer Institute; West Virginia University; West Virginia University Cancer Institute

**Keywords:** Appalachia, adverse childhood experiences, cancer control, cancer prevention, cancer screening, childhood trauma, health communication, provider relationships

## Abstract

**Introduction:**

Adverse childhood experiences (ACEs) are associated with increased cancer risk. ACEs may affect this risk in a variety of ways, including cancer screening compliance. ACEs can contribute to mistrust in the medical profession, inhibit patient–provider relationships and cause at-risk individuals to miss critical access points to preventive services. Protective factors may play an important role in mitigating ACE-related consequences by supporting resiliency.

**Purpose:**

This study assesses the associations between ACEs, protective factors, patient–provider relationships, stage of cancer at diagnosis, and cancer screening behaviors for West Virginia (WV) cervical cancer survivors.

**Methods:**

WV cervical cancer survivors diagnosed between 2000 and 2020 were mailed a survey which included questions on demographic information and cancer screening behaviors, alongside three scales to measure depth of patient–provider relationships, ACEs, and protective factors.

**Results:**

Ninety participants completed the survey. ACEs were associated with weaker patient–provider relationships (*p* < .01) and fewer protective factors (*p* < .01). More protective factors were associated with stronger patient–provider relationships (*p* < .01), earlier stage of cancer at diagnosis (*p* < .05) and positive cancer screening behaviors. Positive cancer screening behaviors were associated with deeper patient–provider relationships (*p* < .05). A statistically significant model (*p* = .004) using ACE and resilience scores was able to account for 13% of the explained variability in depth of patient–provider relationships.

**Implications:**

These findings suggest an important interplay between ACEs, protective factors, and patient–provider relationships on cancer screening behaviors. Future studies should consider these variables in different populations. In addition, interventions focused on enhancing patient–provider relationships and supporting acquisition of protective factors should be considered.

## INTRODUCTION

Adverse childhood experiences (ACEs), also referred to as childhood trauma, are adult-perpetrated negative events that occur early in life, prior to age 18 years.^1^ These traumatic events can include abuse, neglect, and a range of household dysfunction, such as witnessing violence, growing up with familial discord, and living with an individual who had substance abuse or mental health challenges.^1^–^3^ ACEs are associated with a range of negative health outcomes across the lifespan,^1^–^3^ including an increased risk of cancer.^1^,^2^ While the association between ACEs and increased cancer risk is not fully understood,^2^ identifying the nature of this relationship is important for upstream cancer prevention efforts.^4^

ACEs may increase cancer risk in a variety of ways.^2^ This increased risk is associated with cancer in adulthood, not childhood cancer, indicating that factors contributing to disease progression are key to understanding this relationship.^5^ Strong associations between ACEs and factors such as obesity, alcohol use, and tobacco use have been identified, suggesting they play an important role in understanding increased cancer risk in this population.^4^

A growing body of literature suggests that childhood trauma may negatively affect cancer screening compliance—defined as receiving recommended, routine, on-time cancer screenings—which could lead to poor health outcomes.^2^,^6^–^9^ ACEs, particularly sexual abuse, have been shown to be associated with cervical cancer screening behaviors.^8^ Emerging evidence has linked childhood trauma with colorectal and breast cancer screening, as well.^6^,^7^ As cancer screenings are important tools used to reduce mortality through early detection of disease, understanding the drivers of these associations are important to the development of interventions. Cancer survivors have a higher rate of screening compliance compared to the general population.^10^ Screening in this population is particularly important, as they are at an increased risk for future cancers.^11^ The effect of ACEs on the screening behaviors of cancer survivors has not been explored, indicating the need for research.

Primary care providers serve as an important source of knowledge about cancer and their recommendation is critical to patient screening compliance.^11^ Without this established relationship, individuals may miss important encouragement to engage in life-saving preventive services.^11^ Trusting relationships with providers are associated with regular cancer screening compliance and potentially earlier detection of cancer,^12^ suggesting the important role of this relationship in improving mortality rates. Individuals who have experienced childhood trauma may have difficulty sustaining long-term relationships with primary care providers.^13^ Attachment Theory is a useful theoretical framework to understand patient–provider relationships within the context of ACEs. This theory postulates that an individual’s attachment style, which is thought to be developed with early caregivers, strongly influences how they relate to others.^14^,^15^ Previous findings suggest that childhood trauma is associated with less patient compliance^15^ and greater medical mistrust.^14^ This mistrust may affect the quality of patient–provider relationships and lead to poorer health outcomes across the lifespan,^15^ also suggesting the need for further research.

Resilience describes the process by which positive outcomes occur despite negative experiences and adversity.^16^ While ACEs have been shown to have a negative impact across the lifespan,^1^ protective factors may mitigate these potentially deleterious effects by increasing resiliency.^17^,^18^ Protective factors are characteristics of positive individual, family, or community relationships and are associated with improved outcomes.^18^ These contrast with risk factors, which are the negative characteristics of individual, family, or community relationships and are associated with poorer outcomes.^18^ Many protective factors focus on providing a safe, nurturing, and stable environment, both within the family and within the broader community.^17^,^18^ Individuals with increased childhood trauma were less likely to report mental and physical distress when they identified the presence of protective factors in their lives, highlighting the potentially mediating effect on the consequences of ACEs.^17^,^18^ Research is needed to understand how protective factors can be used to develop interventions to reduce cancer risk.^2^

Individuals in rural areas may be prone to having increased levels of ACEs.^19^ As ACEs are associated with areas that have high social and economic stressors,^20^ exploring specific populations within rural areas is warranted. Appalachia is a largely rural region of the U.S. and is characterized by a wide array of social challenges, health inequities, and disparities.^22^ These challenges may be associated with the intergenerational economic hardships of the region.^20^,^21^ Appalachia has increased levels of substance abuse, lung cancer, heart disease, tobacco use, and mental health disorders compared to non-Appalachian rural areas, suggesting a potentially higher level of ACEs in the population.^20^,^21^ Overall, Appalachia has poor cancer-related outcomes across the continuum.^20^ Mortality rates are significantly higher in this region, highlighting suboptimal cancer screening behaviors.^22^ This interplay of social and economic challenges in the region suggest that ACEs may play an important role in poor health outcomes^22^ and warrants further study.

## PURPOSE

This study seeks to understand associations between patient–provider relationships, childhood trauma, protective factors, and cancer screening behaviors in the context of rural Appalachian cancer survivors. To date, the clearest association between ACEs and cancer screening behaviors is with cervical cancer. This study builds on this existing foundation through a focus on cervical cancer survivors in rural Appalachia. Attachment Theory is used as the theoretical framework from which to understand the impact of childhood trauma on patient–provider relationships. The purpose of this study is three-fold:(1) to understand the associations between patient–provider relationships, ACEs, protective factors, and cancer screening behaviors in Appalachian cervical cancer survivors; (2) to assess the associations between patient–provider relationships, ACEs, protective factors, and the stage of cancer at diagnosis in Appalachian cervical cancer survivors; and (3) to determine if ACEs and protective factors could predict the depth of patient–provider relationships.

## METHODS

### Study Sample

A purposeful sampling strategy was used to identify study participants. As West Virginia (WV) is the only state entirely located within the Appalachian Region, it served as the focus of study recruitment. All WV cervical cancer survivors aged 18 years or older with a diagnosis between 2000 and 2020 were included in the participant pool. The West Virginia Cancer Registry (WVCR) developed a list of 1,137 eligible participants to be sent a 58-item survey. No incentive was offered for participation. Of this participant pool, 90 participants completed the survey and returned it via mail or using the online Qualtrics option, representing an 8% response rate. This sample size provided a 10% margin of error and a 95% confidence interval for analysis. Characteristics of the participants included in the study can be found in Table 1.

### Procedures

A cross-sectional survey research design was used for the study. The research team sent study materials to the WVCR. WVCR staff mailed the study materials, which included a cover letter, informed consent, survey instrument, and a prepaid return envelope, to all 1,137 eligible participants. The cover letter provided a link to access the survey via Qualtrics, if the participant preferred to complete the survey online. Two weeks after the initial mailing, WVCR staff mailed a reminder letter to all eligible participants. The initial mailing took place in July 2021 with the reminder letter sent in August 2021. Data collection ended by September 2021. West Virginia University Institutional Review Board approved the study protocol (IRB #2101203926).

### Measures

Participants completed a survey which collected basic demographic information for characterization, general cancer diagnosis information, cancer screening behavior information, and answers along three scales, which measured depth of patient–provider relationships, childhood trauma, and protective factors. Cancer screening behavior questions (scored “yes” = 1 or “no” = 2) included asking if the participant currently had a primary care provider, if they spoke with their provider about cancer screening, and if they were current with cancer screenings. General cancer diagnosis information included collecting the stage and age at cancer diagnosis.

ACEs were measured using the Adverse Childhood Experiences Questionnaire, which included 10 items that assessed for childhood trauma, including abuse (physical, emotional, and sexual), household dysfunction, and neglect,^1^ (e.g., “[d]id you live with anyone who was a problem drinker or alcoholic or who used street drugs?”). For each question to which a participant answered “yes,” 1.00 was added to the potential ACE score. Total ACE scores could range from 0 to 10, with higher scores indicating an increased number of ACEs.

Protective factors were measured using the 14-item Resilience Questionnaire, which included 14 items that mirrored the ACEs Questionnaire,^24^ (e.g., “I believe that my mother loved me when I was little.”). Each of the items were answered using a Likert scale ranging from “definitely not true” to “definitely true.” For each question that a participant answered as “definitely true” or “probably true,” 1.00 was added to the resilience score. Total resilience scores could range from 0 to 14, with higher scores indicating an increased number of protective factors.

The patient–provider relationship was measured using the Patient–Doctor Depth-of-Relationship Scale, which included eight items,^25^ (e.g. “[t]his provider knows me as a person.”) This scale measures patient perceptions of their relationship with their provider, including feelings of being heard and understood.^25^ Each of the items were answered using a Likert scale ranging from “disagree” to “totally agree.” Participants had the opportunity to score from 0 (selecting “disagree”) to 4.00 (selecting “totally agree”) for each item, with total scores ranging from 0 to 32. Higher scores indicated a deeper relationship between patients and providers.

### Statistical Analyses

Descriptive statistics were used to characterize the survey population. Frequencies were run for all study variables, including mean and standard deviation for study scales. A Spearman’s rank-order correlation was run to assess the associations between the continuous variables of ACE score, resilience score, and the depth of patient–provider relationship score. A point-biserial correlation was run between the three study scales and cancer screening behaviors in WV cervical cancer survivors. Chi-squared tests of association were run between the noted cancer screening behaviors. A Spearman’s rank-order correlation was run to assess the associations between study scales and the stage of cervical cancer at diagnosis. A rank biserial correlation was run between the stage of cervical cancer at diagnosis and cancer screening behaviors. Finally, a multiple regression was run to determine if ACE and resilience scores could predict the depth of patient–provider relationships.

## RESULTS

Table 2 illustrates the associations between all study variables. Relationships between all the study scales were statistically significant at the *p* < .01 level. Higher ACE scores had a negative association with the depth of patient–provider relationship scores and a negative association with resilience scores. Increased resilience scores had a positive association with the depth of patient–provider relationships. Conversely, increased protective factors were associated with a stronger patient-provider relationship.

Increased resilience scores were statistically significantly associated with having a regular primary care provider, speaking with providers about cancer screenings, and being current with cancer screenings. Higher ACE scores showed a statistically significant association with not being current with cancer screenings. Depth of patient–provider relationship had a statistically significant association with talking with a provider about cancer screening and being current with cancer screenings. There was a statistically significant association between talking with a provider about cancer screenings and being current with cancer screenings, χ^2^(1) = 4.511, *p* = .034. In addition, there was a statistically significant association between having a regular primary care provider and being current with cancer screenings, χ^2^(1) = 4.520, *p* = .034. The stage of cancer at diagnosis had a statistically significant association with the resilience score. In addition, the stage of cancer at diagnosis had a statistically significant association with being current with cancer screenings, ϕ = .220, *p* = .043.

Table 3 illustrates the results of a multiple regression model looking at the effect of protective factors and childhood trauma on patient–provider relationships. This model statistically significantly predicted the depth of patient–provider relationships, *F*(2,82) = 5.936, *p* = .004, adj.*R*^2^ = .105. Neither coefficient added statistically significantly to the prediction independently, *p* = .090 (resilience score) and *p* = .060 (ACE score). Taken together, ACE and resilience scores accounted for 13% of the explained variability in the depth of patient–provider relationships.

## DISCUSSION

This study sought to understand associations between childhood trauma, patient–provider relationships, protective factors, and cancer screening behaviors among WV cervical cancer survivors. In addition, the research team sought to understand if study scales were associated with the stage of cervical cancer at diagnosis. Finally, this study sought to understand if ACE scores and resilience scores could be used to predict depth of patient–provider relationships. While existing research identified associations between different types of cancer screening and childhood trauma,^2^,^6^–^9^ no previously identified studies examined this in the context of cancer survivors or assessed for protective factors concurrently.

The association between patient–provider relationships, ACE scores, and resilience scores were statistically significant, showing an important interplay among these variables. As ACE scores rose in the survey population, protective factors and depth of patient–provider relationships decreased. As protective factors rose, ACE scores decreased, and depth of patient–provider relationships increased. This supports previous research that suggested protective factors may play an important mitigating role to childhood trauma.^17^,^18^ Looking through the lens of Attachment Theory, this suggests that while ACEs may contribute to mistrust of the medical profession,^16^ this can potentially be mitigated through an increase in protective factors, offering an important avenue for intervention development.

While higher ACE scores were associated with not being current with cancer screenings, which supported previous findings,^2^,^6^–^9^ protective factors had a stronger association on the assessed cancer screening behaviors. Again, this may support the importance of protective factors as critical to the mitigation of the long-term effects of childhood trauma.^17^,^18^ While the ACE score and the depth of patient–provider relationship score did not show a statistically significant association with the stage of cervical cancer at diagnosis, the resilience score did. Earlier diagnosis of cancer was associated with an increased number of protective factors. Future research should explore how to enhance protective factors in at-risk populations. Novel approaches such as adaption-based resiliency offer opportunities for intervention development. Furthermore, longitudinal studies should explore these associations over time.

The depth of patient–provider relationships did show an association with having cancer screening conversations and overall screening compliance, which aligns with previous findings related to Attachment Theory.^14^ As noted by Dr. John Bowlby, attachment patterns are developed early in life and maintained later in adulthood.^25^ If these attachments are insecure due to childhood trauma, patient–provider relationships in adulthood could be problematic, as anxiety would not be sufficiently assuaged by the interaction, which could affect health care.^26^ Study findings seem to support this, as having conversations with providers was associated with being current with cancer screenings. This supports previous findings suggesting the importance of provider recommendation for screening completion.^11^

Individuals with a history of childhood trauma have a greater reliance on emergency medical services, so they may not have regular access to cancer screening, potentially affecting long-term health outcomes.^6^–^8^ In this study population, there was a statistically significant association between being current with cancer screening and previous early detection of cancer, reinforcing the importance of regular preventive services to positive health outcomes. Establishing meaningful relationships with providers may be an important intervention for at-risk populations to increase utilization of preventive services.

Future research should explore strategies to encourage and strengthen the development of patient–provider relationships in the primary care setting. This would be particularly important in at-risk populations that may have higher levels of childhood trauma.

The model developed through this study showed that childhood trauma and protective factors may contribute to the quality of patient–provider relationships. ACEs may negatively affect the depth of this relationship, as characterized through Attachment Theory, and protective factors may facilitate it. Considering one factor or the other independently may not capture the full picture. While this model found that each coefficient was not statistically significant, collectively they formed a statistically significant model that warrants further study. Findings support previous research that described the important role providers play in patients’ cancer screening choices,^11^ this suggests that understanding what affects this relationship would be important for intervention development.

This study examined a specific population, cervical cancer survivors from within the Appalachian Region. Social and economic factors suggest a high prevalence of ACEs, making the findings critical to the reduction of health disparities in this region. Cancer survivors have higher rates of cancer screening compliance compared to the general population,^10^ highlighting that the associations described in this study may also be important to explore in population that screen for cancer at a lower rate. Future research should consider these variables in different groups of cancer survivors and in representative general populations to assess if these associations are more generalizable.

### Limitations

While this study adds to the limited research in this area, it is not without its limitations. The return rate of the mailed survey was low, not achieving the goal of 20% participation. The sensitive nature of the survey, particularly the ACE-related questions, may have inhibited returns. Response bias is a potential limitation with most survey research and may have affected participants in this study. These findings are self-reported measures and not clinical data, which also limits the study. As this was a cross-sectional study design, change in relationship among these variables over time could not be assessed. In addition, the population is not representative of cancer survivors or cervical cancer survivors, so results are not able to be generalized. The population in WV is largely white, aligning with study respondents, but did not provide racial and ethnic diversity. Finally, this study did not assess for healthcare service access from participants, which has been identified as an important consideration for this population in previous studies;^6^–^8^ instead it focused on patient–provider relationships and cancer screening behaviors.

## IMPLICATIONS

Study findings suggest that there is an interplay between protective factors, childhood trauma, and patient–provider relationships and that they may be associated with cancer screening compliance and long-term health outcomes. These findings provide insights to health professionals as they work to build relationships with patients from at-risk populations. In addition, these considerations may be important for upstream cancer prevention efforts, including for health policy. Increasing resiliency through protective or promotive factors across systems, including families, communities, healthcare organizations, and economies, offer critical policy change opportunities that may help improve long-term health outcomes.

This exploratory study provides formative research in an area with limited literature. It provides multiple avenues of future research and potential interventions which could have a far-reaching effect on primary care delivery. Future research should explore these factors in different populations, including cancer survivors more broadly and the general population. In addition, interventions focused on developing protective factors for at-risk populations and enhancing patient-provider relationships should be considered. Furthermore, this study provides a framework from which to guide future research in the area, including study scales and the use of Attachment Theory to understand patient–provider relationships related to childhood trauma.

**SUMMARY BOX**

**What is already known about this topic?**

Adverse childhood experiences (ACEs) are traumatic events that occur in childhood that increase risk for chronic disease in adulthood, including cancer. This increased cancer risk may occur for many different reasons, including changes in screening practices.

**What is added by this report?**

The study team identified an interplay between ACEs, protective factors, and patient-provider relationships on cancer screening behaviors and stage of diagnosis. This is a particularly important finding for Appalachia as social and economic factors suggest a high prevalence of ACEs in the region.

**What are the implications for future research?**

These findings provide health professionals insights to an at-risk population which may affect patient–provider relationships and cancer screening compliance. Future research should expand on these variables in more diverse populations an seek to identify and develop intervention and policy recommendations.

## Figures and Tables

**Table 1:** Survey participant characteristics (n = 90)

Characteristics	Percent
**Age**	
26–40 years	10.5%
42–65 years	60.5%
66 years and older	22.1%
None given	7.0%
**Education level**	
Some high school	5.8%
High school diploma/GED	39.5%
Some college/technical degree	25.6%
College degree	22.1%
Advanced degree	7.0%
**Race**	
White	100%
**Ethnicity**	
Hispanic/Latino	2.3%
Not Hispanic/Latino	97.7%
**Marital status**	
Married	57.0%
Separated/divorced/widowed	32.6%
Never married	8.1%
Partnered	2.3%

**Table 2:** Associations between study scales, stage of cancer at diagnosis, and cancer screening behaviors

Variables	Frequencies			Depth	ACE	Resilience
**Scales**	Range	Mean	SD			
Patient-provider relationship score (Depth)	0–32	23.44	8.42	–	−.285†	.325†
ACE score (ACE)	0–10	1.97	2.10	−.285†	–	−.438†
Resilience score (Resilience)	0–14	11.60	2.76	.325†	−.438†	–
**Cancer diagnosis**	Range	Mean	SD			
Stage of cancer diagnosis	1–4	2.67	1.59	−.080	.029	−.233*
**Cancer screening behaviors**	Range	Yes %	No %			
Regular primary care provider	1–2	94.1%	5.9%	−.178	.093	−.249*
Talk with provider about cancer screening	1–2	75.3%	24.7%	−.261*	.136	−.316†
Current with cancer screenings	1–2	80.8%	19.2%	−.224*	.214*	−.295†

NOTES:

* *p* < .05

† *p* < .01

**Table 3:** Model for depth of patient–provider relationship using childhood trauma and resiliency coefficients

Patient-provider relationship*	*B*	95% CI for *B*		*SE B*	β	*R* ^2^	Δ*R*^2^
		*LL*	*UL*				
**Model**						.13†	.11†
Constant	18.22†	9.19	27.24	4.54			
ACE score	.873	1.78	.04	.458	.220		
Resilience score	.593	.09	1.28	.345	.198		

NOTES:

* Model = “Enter” method in SPSS; *B* = unstandardized regression coefficient; CI = confidence interval; *LL* = lower limit; *UL* = upper limit; *SE B* = standard error of the coefficient; β = standardized coefficient; *R*^2^ = coefficient of determination; Δ*R*^2^ = adjusted *R*^2^.

† *p* < .05
